# TPGLDA: Novel prediction of associations between lncRNAs and diseases via lncRNA-disease-gene tripartite graph

**DOI:** 10.1038/s41598-018-19357-3

**Published:** 2018-01-18

**Authors:** Liang Ding, Minghui Wang, Dongdong Sun, Ao Li

**Affiliations:** 1 School of Information Science and Technology, University of Science and Technology of China, Hefei, AH230027 China; 2 Centers for Biomedical Engineering, University of Science and Technology of China, Hefei, AH230027 China

## Abstract

Accumulating evidences have indicated that lncRNAs play an important role in various human complex diseases. However, known disease-related lncRNAs are still comparatively small in number, and experimental identification is time-consuming and labor-intensive. Therefore, developing a useful computational method for inferring potential associations between lncRNAs and diseases has become a hot topic, which can significantly help people to explore complex human diseases at the molecular level and effectively advance the quality of disease diagnostics, therapy, prognosis and prevention. In this paper, we propose a novel prediction of lncRNA-disease associations via lncRNA-disease-gene tripartite graph (TPGLDA), which integrates gene-disease associations with lncRNA-disease associations. Compared to previous studies, TPGLDA can be used to better delineate the heterogeneity of coding-non-coding genes-disease association and can effectively identify potential lncRNA-disease associations. After implementing the leave-one-out cross validation, TPGLDA achieves an AUC value of 93.9% which demonstrates its good predictive performance. Moreover, the top 5 predicted rankings of lung cancer, hepatocellular carcinoma and ovarian cancer are manually confirmed by different relevant databases and literatures, affording convincing evidence of the good performance as well as potential value of TPGLDA in identifying potential lncRNA-disease associations. Matlab and R codes of TPGLDA can be found at following: https://github.com/USTC-HIlab/TPGLDA.

## Introduction

Long non-coding RNAs (lncRNAs) are a new class of transcripts, with the length longer than 200nt^1–3^, which have been implicated in a number of normal physiological processes at every stage of life, from embryonic development and cellular cell fate determination to physiological homoeostasis of entire organisms^4^. Accumulating studies have indicated that a large quantity of lncRNAs are critical in many important biological processes such as chromatin modification, transcriptional and post-transcriptional regulation, genomic splicing, differentiation, immune responses, cell cycle control and so on^3,5,6^. Especially, it has been demonstrated that a large number of lncRNAs are involved in numerous complex human diseases^3,4^, such as neurological disorders^7^, coronary artery diseases^8^, cardiovascular diseases^9^, and various cancers^10^. Accordingly, inferring potential associations between lncRNAs and diseases can help us understand the pathogenesis of complex diseases at the molecular level and benefit biomarker identification for disease diagnosis, therapy, prognosis and monitoring^5^.

Up to now, a large amount of lncRNA-related biological data has been distributed in different public databases^11–13^ and only few associations between lncRNAs and diseases have been reported. Collecting and integrating these data from a great number of literatures and databases is costly. Moreover, many biological experiments are time-consuming and expensive. Accordingly, researchers have started to focus on developing computational prediction approaches based on the existing datasets, which can quantify the associations’ probability between lncRNAs and diseases, and the most promising lncRNA-disease associations are used for further biological experimental validation. In this case, the time and cost of biological experiments can be effectively reduced^14^.

In recent years, some computational models have been proposed to identify potential associations between lncRNAs and diseases. For example, based on important assumption that similar diseases are often associated with functionally similar lncRNAs^5^, Chen and Yan propose a computational approach of LRLSLDA to identify potential disease–related lncRNAs. LRLSLDA is a novel semi-supervised learning method in Laplacian regularized least squares framework. Moreover, this method does not require negative samples and can produce reliable results based on lncRNA expression profile and known lncRNA-disease associations. Subsequently, based on the finding that functionally related genes are often associated with phenotypically similar diseases^15,16^, Sun *et al*.^17^. Construct an lncRNA-lncRNA functional similarity network. Then, they develop a global network-based computational approach, RWRlncD, to identify potential lncRNA–disease associations by integrating disease similarity network, lncRNA functional similarity network and experimentally verified lncRNA-disease associations. Based on the same assumption, Ganegoda *et al*.^18^. Further propose a kernel based random walk with restart in heterogeneous network model (KRWRH), which incorporates with lincRNA tissue specific information, disease phenotype information and experimentally validated disease-lincRNA associations. KRWRH uses Gaussian interaction profile kernel to calculate the similarities of diseases and lincRNAs, and random walk with restart method is utilized for final prediction. The good experimental results highlight the importance and effectiveness of inferring potential disease-lincRNA associations using different biological information^18^.

Despite the success achieved by aforementioned methods, another important factor contributing to infer potential lncRNA-disease associations lies in the fact that coding and non-coding genes are often cooperated in human diseases, which has been demonstrated in many previous studies^19–22^ For example, Sahu *et al*.^23^ demonstrate that coding gene-TAF1D and lncRNA-SNHG1 are highly co-expressed in neuroblastoma. At the same time, rich information about gene-disease associations is available in database such as DisGeNET^24^ and PsyGeNET^25^. Therefore, if effectively used, such information may be of great help to infer potential associations between lncRNAs and diseases. Recently, Yang *et al*.^22^ conduct a pioneer study in which the authors integrate coding gene-disease associations and propose a propagation algorithm to infer potential lncRNA-disease associations based on a bipartite graph of coding-non-coding genes-disease. The authors show integrating coding gene-disease associations with lncRNA-disease associations can significantly enhance its prediction performance. Despite its low complexity and effectiveness, the bipartite graph model used in this study treats coding and non-coding genes without distinction and therefore cannot fully account for the heterogeneity of coding-non-coding genes-disease association. In addition, it cannot work for the lncRNAs and diseases without any known associations (hereafter isolated nodes), due to the fact that this method depends on the topological structure of bipartite graph and in consequence isolated nodes cannot get any information^22^. Another pioneer study called ncPred uses a tripartite network to infer potential ncRNA-disease association by integrating ncRNA-target associations and disease-target associations^26^. In this excellent work, the target information gives rise to a bridge connecting the ncRNA and diseases which significantly improve its predictive performance.

Inspired by the above methods, in this paper we present a novel computational approach of a Tripartite Graph for potential LncRNA-Disease Association identification (TPGLDA) by integrating gene-disease associations and lncRNA-disease associations. The method begins with an lncRNA-disease-gene tripartite graph to delineate the heterogeneity of coding-non-coding genes-disease association. Subsequently, an effective resource allocation algorithm is proposed to accurately identify potential lncRNA-disease associations. Furthermore, lncRNA expression similarity and disease semantic similarity are introduced into TPGLDA to make inference for isolated nodes^14,27^. To perform a proper evaluation of our proposed method, we utilize leave-one-out cross validation experiment to demonstrate its superior performance compared with existing approaches. Besides, the analyses of several important cancers (i.e. lung cancer, hepatocellular carcinoma, ovarian cancer, etc.) effectively support the practical application of our method. We then use TPGLDA to infer potential lncRNA–disease associations and some high-ranking results are successfully verified by related literatures and databases, such as Lnc2Cancer^28^, LncRNA2Target^29^ and MNDR^30^. These results afford convincing evidence of the good performance of TPGLDA as well as potential value in supporting further biological experiments and promoting research productivity.

## Results

### Overview of proposed method

**Figure 1:**
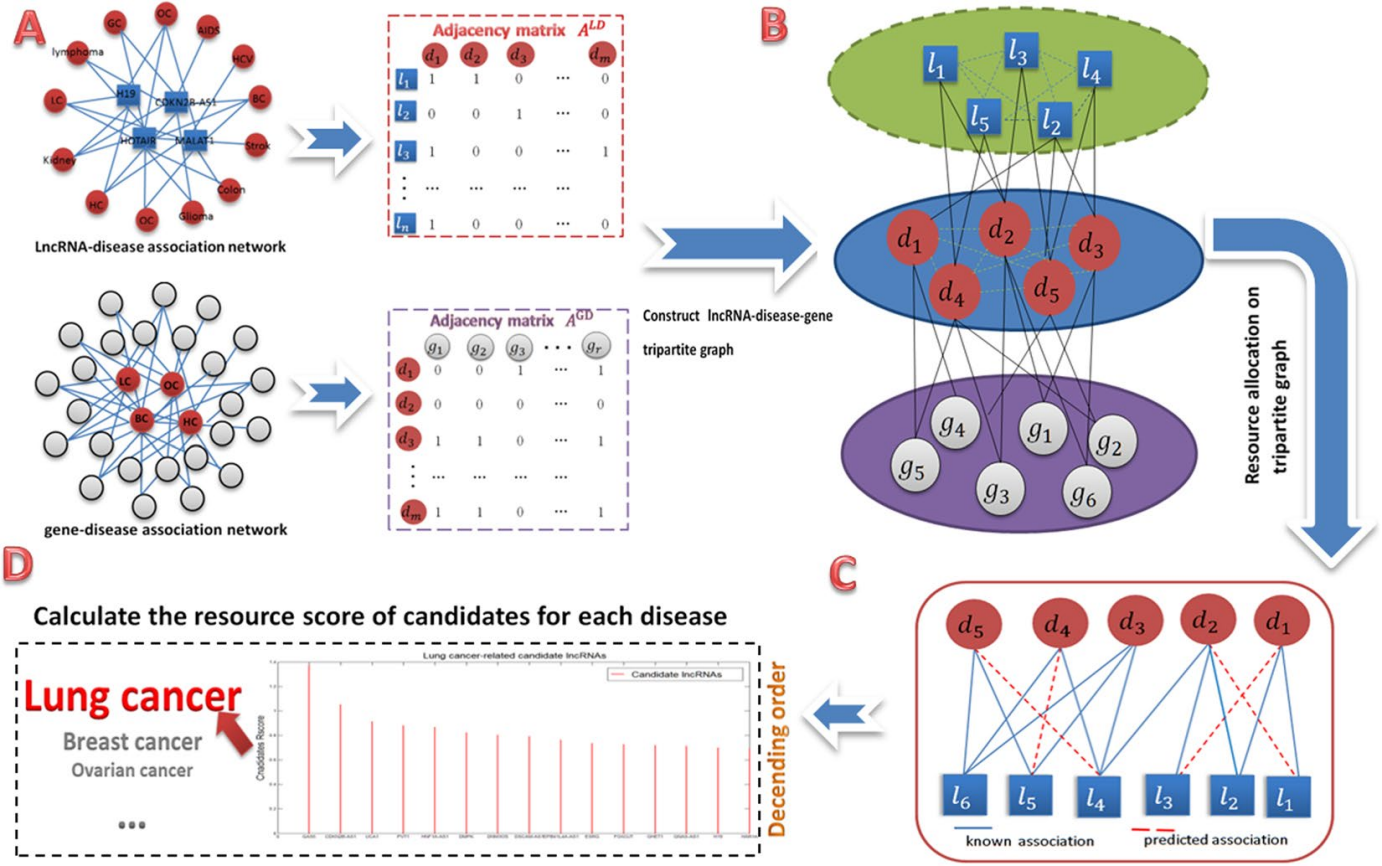
The flowchart of TPGLDA. (**a**) Construct lncRNA-disease and gene-disease adjacency matrix. Calculate interaction profile for isolated nodes and integrate into adjacency matrix for further resource allocation. (**b**) Construct lncRNA-disease-gene tripartite graph. (**c**) Resource allocation on tripartite graph and build the potential lncRNA-disease associations. (**d**)Calculate the resource score (Rscore) of candidate lncRNAs and rank all candidates’ Rscore for each disease in descending order.

The overview of TPGLDA in identifying potential lncRNA-disease associations can be simple summarized as following four steps (Fig. 1). First, we construct lncRNA-disease and gene-disease adjacency matrix by using known lncRNA-disease associations and known gene-disease associations. For an isolated node, we need to calculate the interaction profile (Eq. 12) and then integrate this vector into adjacency matrix for further resource allocation. Second, we construct lncRNA-disease-gene tripartite graph. Third, the process of resource-allocation on tripartite graph builds the potential lncRNA-disease associations. Finally, the resource score (Rscore) of each potential disease-related candidate lncRNA is calculated in turn. We rank all candidates’ Rscore for each disease in descending order, and a higher score will have greater possibility for further verification.

### Evaluation of prediction performance

Leave-one-out cross validation (LOOCV) is implemented on our gold standard dataset to evaluate the performance of TPGLDA in inferring potential associations between lncRNAs and diseases^5,18,22^. At each step of the LOOCV experiment, each known lncRNA-disease association is removed from the training samples in turn as test sample, whereas other known associations are taken as training samples for model learning^5,14,22^. Then, the receiver operating characteristics (ROC) curve is utilized to demonstrate the predictive performance of our proposed method and other methods mentioned in this paper by plotting the true positive rate (Sn, sensitivity) and the false positive rate (1-Sp, 1-specificity) at different cutoff points. Here, sensitivity refers to the ratio of positive cases which can be correctly identified, and specificity represents the percentage of negative cases which can be correctly predicted. The value of AUC is calculated from the corresponding area under ROC curve, and the perfect performance appears in AUC = 1 while the random performance emerges in AUC = 0.5^14,31,32^. Besides, we also adopt other evaluation measures such as accuracy (Acc), precision (Pre), and Matthews’s correlation coefficient (MCC)^31,33^. The definitions can be obtained as follows:

1 $$Sensitivity=\frac{TP}{TP+FN}\,$$

2 $$Specificity=\frac{TN}{TN+FP}\,$$

3 $$Accuracy=\frac{TN+TP}{TN+TP+FN+FP}\,$$

4 $$Precision=\frac{TP}{TP+FP}\,$$

5 $$Matthews\mbox{'}s\,correlation\,coefficient=\frac{TP\times TN-FP\times FN}{\sqrt{(TP+FN)\times (TP+FP)\times (TN+FN)\times (TN+FP)}}$$

where TP means true positives, FP refers to false positives, TN is true negatives, and FN represents false negatives.

### Compared with other methods

**Figure 2:**
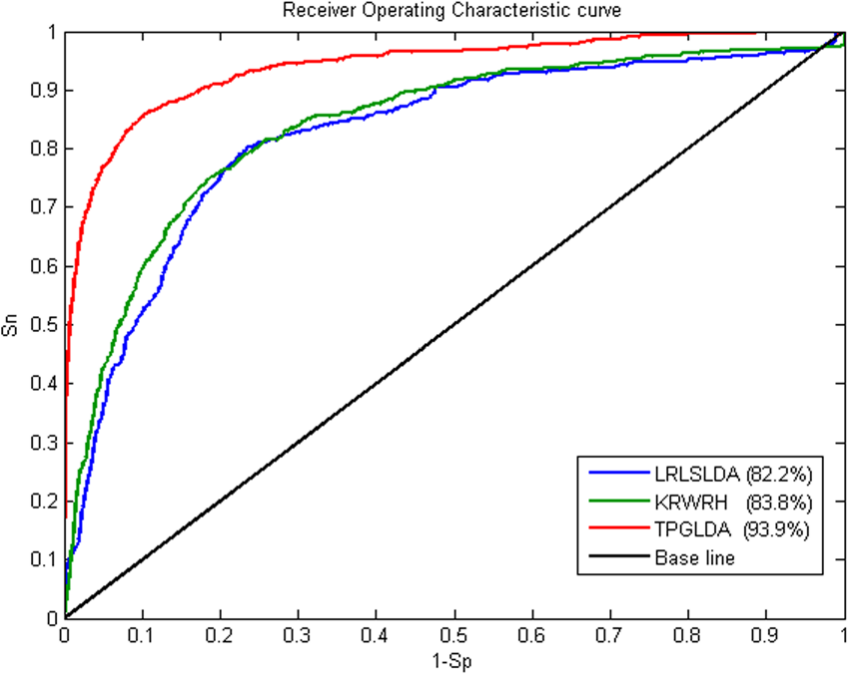
Performance comparison between TPGLDA, LRLSLDA and KRWRH in terms of AUC and ROC curve based on LOOCV. As a result, TPGLDA achieves the highest AUCs of 0.939. The base line indicates random performance.

In order to comprehensively assess the predictive ability of TPGLDA to predict lncRNA–disease associations, we compare our method with two state-of-the-art methods: LRLSLDA^5^ and KRWRH^18^, and the corresponding ROC curves of different methods are shown in Fig. 2. Note that Yang’s method^22^ is not assessed here as it requires the node degree of each candidate ≥2. As a result, both LRLSLDA and KRWRH achieve reliable performance with AUC values of 82.2% and 83.8%, respectively, and TPGLDA has improved with an AUC value of 93.9%. Besides, other common performance evaluation measures, including *Sn*, *Sp*, *Pre*, *Acc*, and *MCC*, are also used to measure the predictive performance of these methods, and the results are shown in Table 1. Here, we adopt two stringency levels to measure the predictive performance^31,34^. At medium stringency level of specificity (Sp = 95.0%), KRWRH achieves the values of *Sn*, *Pre*, *Acc*, and *MCC* are 42.6%, 18.7%, 93.6% and 25.4%, respectively, which performs slightly better than LRLSLDA. By contrast, the corresponding values of TPGLDA are 76.8%, 29.4%, 94.5%, 45.4%, respectively. When the stringency level of specificity enlarges to 99.0%, the performance of our proposed method and the other two methods are consistently improved across all measures. Overall, these assessments generally confirm the good performance of TPGLDA in recovering experimentally verified lncRNA-disease associations.

**Table 1:** Comparison with other computational approaches at two stringency levels (*Sp* = 99.0% and *Sp* = 95.0%).

	TPGLDA	KRWRH	LRLSLDA
*Sp*=99.0%			
*Sn*	53.5%	11.7%	10.7%
*Acc*	97.8%	96.7%	96.7%
*Pre*	59.2%	24.1%	22.6%
*MCC*	55.2%	15.2%	14.0%
*Sp*=95.0%			
*Sn*	76.9%	42.6%	35.2%
*Acc*	94.5%	93.6%	93.4%
*Pre*	29.4%	18.7%	16.0%
*MCC*	45.4%	25.4%	20.7%

**Figure 3:**
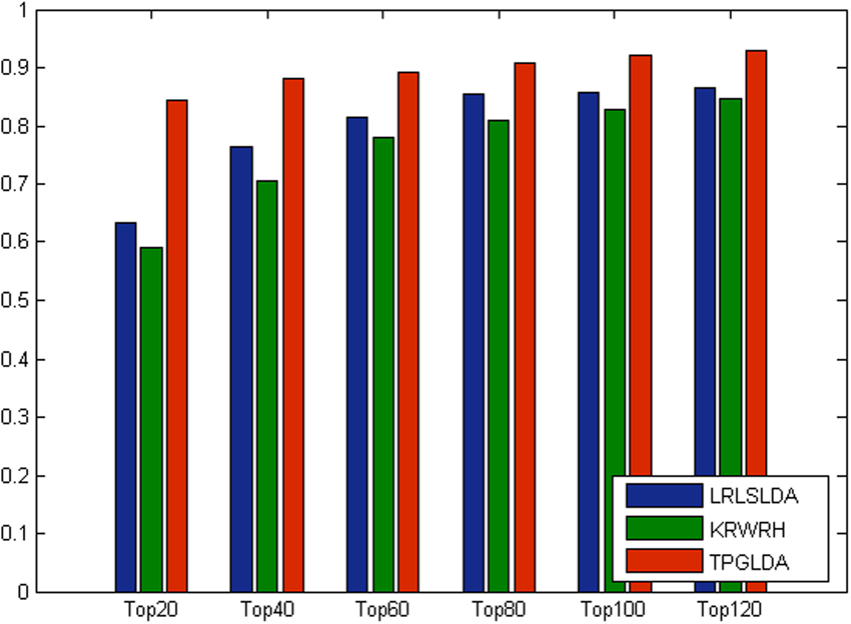
The average AUCs across all the diseases at different top *k* cutoffs.

**Figure 4:**
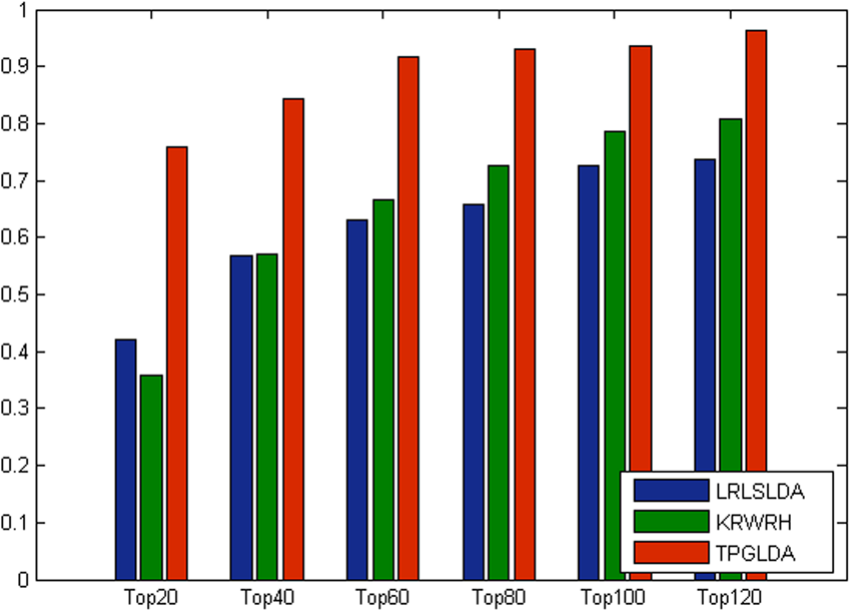
The average recall across all the diseases at different top *k* cutoffs.

Furthermore, considering the importance of top portion of the prediction results^32^, the corresponding AUC values within the top *k* candidates of ranking lists are measured and the detailed results are shown in Fig. 3. In the top 20 ranking lists, the corresponding AUC values achieved by LRLSLDA and KRWRH are 63.3% and 58.9%, respectively, whereas TPGLDA achieves an AUC value of 84.4%. For the results in the top 100, LRLSLDA achieves good performance with an AUC of 85.6%. By contrast, TPGLDA obtains a better AUC value of 92.2%. Besides, we also report the corresponding recall rate (Fig. 4), which measures the number of known lncRNA-disease association pairs that can be correctly identified within the top *k* candidates of ranking lists^31,32^. In the top 20 candidates, our method can successfully rank about 76% of known lncRNA-disease association pairs. When the rank threshold reaches to 100, the recall values of LRLSLDA and KRWRH are improved to 72.6% and 78.7%, respectively, and the corresponding value obtained by TPGLDA is 93.6%. Taken together, TPGLDA achieves decent recall in above different top *k* ranking lists, suggesting that our method can infer the largest number of positive samples based on different cutoffs.

Similar to previous studies^31,32^, we apply the LOOCV experiment on 15 diseases for demonstrating the practical predictive ability of different methods, and the corresponding AUC values are shown in Table 2. As a result, TPGLDA compares favorably with KRWRH and LRLSLDA in terms of AUC values. For example, for bladder cancer, KRWRH and LRLSLDA achieve the corresponding AUC value of 77.4% and 76.5%, respectively, and in comparison TPGLDA obtains an AUC value of 88.3%. Also, the AUC value of TPGLDA for breast cancer reaches 85.2%, which is more than 15% better than the other methods investigated in this paper. Furthermore, the average AUC values of TPGLDA, KRWRH and LRLSLDA for all 15 diseases are calculated, and the results are 87.4%, 76.7% and 74.1%, respectively. Besides, we further report the Friedman rank sum test on our dataset to show the statistical significance in performance improvement of TPGLDA (Supplementary Table S6). These examinations demonstrate that TPGLDA has practical ability to predict various potential lncRNA-disease associations.

**Table 2:** Prediction results for TPGLDA, KRWRH and LRLSLDA utilizing leave-one-out cross validation experiment on 15 diseases.

Disease name	No. of Associated lncRNAs	AUC		
		TPGLDA	KRWRH	LRLSLDA
Gastric Cancer	24	0.893	0.832	0.756
Colorectal Cancer	21	0.884	0.782	0.687
Breast Cancer	20	0.852	0.675	0.655
Hepatocellular Carcinoma	20	0.911	0.891	0.751
Non-Small Cell				
Lung Cancer	15	0.799	0.759	0.765
Prostate Cancer	13	0.886	0.807	0.758
Esophageal Squamous				
Cell Carcinoma	13	0.822	0.835	0.739
Ovarian Cancer	12	0.892	0.731	0.768
Bladder Cancer	11	0.883	0.774	0.765
Lung Cancer	9	0.828	0.737	0.750
Melanoma	9	0.939	0.627	0.815
Glioma	9	0.820	0.710	0.808
Tumor	8	0.950	0.786	0.625
Schizophrenia	8	0.860	0.854	0.630
Papillary Thyroid Carcinoma	7	0.892	0.700	0.835

In addition to our gold dataset used in performance evaluation, Chen *et al*. (2013)^35^ dataset used in ncPred^26^ is applied to make comparison with ncPred (Supplementary Table S7). By applying a 10-fold cross-validation procedure repeated 30 times, we calculate the corresponding averaged AUC value and the result obtained by TPGLDA is 0.7586 ± 0.0306, which is comparable with the result (0.7566 ± 0.0218) reported by ncPred^26^. At the same time, the Friedman rank sum test’s result is also reported in Supplementary Table S8. The above two results indicate that there is no statistically significant difference between ncPred and TPGLDA predictions in Chen *et al*. dataset.

At the same time, the time complexity of resource allocation in lncRNA-disease association and gene-disease are *O*(*n*^2^*m*) and *O*(*nrm*), respectively. Considering the fact that the number of disease-related genes is an order of larger than the number of lncRNAs and diseases, the overall time complexity of resource allocation in tripartite graph is *O*(*nrm*), which is more efficient to that of ncPred (*O*(*r*^2^*m*))^26^. However, the parallelization and optimization techniques can greatly reduce the differences in computational speed. Also, we compare the running time of different methods and the results show the running time of TPGLDA is 0.6 second in average, which is comparable with other methods (Supplementary Table S9).

### Case studies

In addition to LOOCV experiment, we also employ TPGLDA to rank all candidate lncRNAs investigated in this paper, and these predictions are used for further analysis in this study. In consistence with previous studies^5,14,22^, all experimentally validated lncRNA-disease associations are utilized as training sample. Subsequently, the resource score for each potential lncRNA-disease association is calculated in turn, and the predicted results are listed in Supplementary Table S3 in descending order. Higher *Rscore* indicates greater potential association between lncRNA and disease. In order to further verify the ability of TPGLDA in discovering potential lncRNA–disease associations, the case studies of three diseases: hepatocellular carcinoma, lung cancer and ovarian cancer are reviewed in detail. Here, the top 5 predicted disease-related lncRNAs computed by TPGLDA and their evidences are listed in Table 3. Predictive results are supported by relevant literatures and databases, and the detailed cases can be interpreted by the examples as follow.

**Table 3:** The top 5 predictions computed by TPGLDA for Lung Cancer, Hepatocellular Carcinoma and Ovarian Cancer and the confirmation for their associations by related databases.

LncRNA	TPGLDA’s rank	Evidences (PMID)	Description
**Lung Cancer**			
GAS5	1	25925741,24357161	Lnc2Cancer,LncRNA2Target
CDKN2B-AS1	2	21489289,26408699	MNDR,Lnc2Cancer
UCA1	3	26380024	Lnc2Cancer
PVT1	4	26493997;26493997	Lnc2Cancer,literature
HNF1A-AS1	5	25863539	literature
**Hepatocelluar Carcinoma**			
GAS5	1	26404135, 26163879	Lnc2Cancer, literature
SOX2-OT	2	26097588	Lnc2Cancer
PVT1	3	25624916	Lnc2Cancer
LINC00152	4	27351280, 26356260	Lnc2Cancer, literature
UCA1	5	27215316, 27167190	Lnc2Cancer, literature
**Ovarian Cancer**			
MEG3	1	24859196	Lnc2Cancer,LncRNA2Target
GAS5	2	26503132	Lnc2Cancer
CCAT2	3	27283598	Lnc2Cancer
BANCR	4	unconfirmed	unconfirmed
CDKN2B-AS1	5	27095571	Lnc2Cancer

Lung cancer is one of the most common cancers worldwide which has extremely high mortality rate^36^. In the United States, lung cancer ranks second only to the highest cancer deaths according to the Estimated New Cancer Cases of the ten leading cancer types^37^. Among the top 5 lung cancer-related candidates ranked by TPGLDA, all 5 potential lncRNAs are verified to be associated with lung cancer by related literatures and databases. For example, the lncRNA-CDKN2B-AS1 promotes NSCLC cell proliferation and inhibits apoptosis by suppressing KLF2 and P21 expression^38^. Furthermore, recent research demonstrates that upregulated lncRNA-UCA1 contributes to progression of lung carcinoma, and lncRNA-UCA1 holds great promise as a potential predictive biomarker in clinical diagnosis for lung cancer^39^.

Hepatocellular carcinoma is predominant component of the primary liver cancer, which is the fifth most common cancer around the world as well as the third most common cause of cancer mortality^40^. The top three hepatocellular carcinoma-related candidates, lncRNA-GAS5, lncRNA-SOX2-OT and lncRNA-PVT1, are all confirmed by recent experimental reports. LncRNA-GAS5, acting as a proto-oncogene, is revealed to be critical to hepatocellular carcinoma and the deletion allele is significantly correlated with higher expression of lncRNA-GAS5 in hepatocellular carcinoma tissues^41^. Besides, the up-regulation of lncRNA-SOX2-OT is reported to facilitate hepatocellular carcinoma cell metastasis and high expression of lncRNA-SOX2-OT is demonstrated to be associated with histological grade, TNM stage and vein invasion^42^. Furthermore, Ding *et al*.^43^ point out that lncRNA-PVT1 is associated with tumor progression and can serve as a novel biomarker for predicting tumor recurrence in hepatocellular carcinoma-related patients.

Ovarian cancer is a major case of cancer deaths in women, especially for ovarian epithelial carcinoma^44,45^. Currently, this cancer is generally detected in the late diagnosis, and the etiology of ovarian cancer is poorly understood for us^45^. Among the top 5 predictions of ovarian cancer, most lncRNAs can be confirmed by related literatures. For example, a recent experimental result shows that lncRNA-MEG3 may play a significant role as a tumor suppressor in ovarian cancer cells^46^. Furthermore, LncRNA-GAS5 is verified to be related to ovarian cancer cell apoptosis by means of the mitochondria-mediated apoptosis pathway, which can be used as a new therapeutic target and has an important role in disease progression^47^.

In addition to the above mentioned diseases, TPGLDA also achieves decent prediction results for other diseases. For examples, lncRNA-TUG1 is ranked first in our prediction list of colorectal cancer-related lncRNAs, and recent study indicates that the upregulation of lncRNA-TUG1 is closely related to the survival time of colorectal cancer patients^48^. Besides, we find that lncRNA-GHET1 is ranked second in our prediction list, which is shown to be significantly upregulated in colorectal cancer tissues and can serve as a therapeutic target for colorectal cancer expression patterns^49^. As for the prediction list of bladder cancer, lncRNA-PVT1 and lncRNA-ANRIL are ranked first and seventh, respectively, which have been shown to be up-regulated in bladder cancer^50,51^. In addition, LncRNA-CCAT1, ranking fourth in our breast cancer-related prediction list, is found to be up-regulated in breast cancer^52^, and lncRNA-TUG1, ranking first in our predicted list of gastric cancer, can promote the transferring and invading capacity of gastric cancer^53^. The top 10 predictions of these disease-related lncRNAs are listed in Supplementary Table S4. Furthermore, we further use TPGLDA to simultaneously rank all candidate lncRNA-disease associations^5^, and the top 20 potential associations are also shown in Supplementary Table S5. 12 of the top 20 predictions are confirmed by different related databases or literatures. From aforementioned case studies, we find that many predictions are confirmed by recent experimental results. For example, recent study demonstrates that lncRNA-PVT1 (rank 16th) is overexpressed in osteosarcoma and can decrease the survival rate of osteosarcoma patients^54^. Therefore, with the progress of the biological experiments, it is anticipated that more unconfirmed associations in our predictive results will be verified, and those potential lncRNA-disease pairs which have higher ranks will be given reasonable priority for subsequent experimental research. In conclusion, these case studies further suggest that TPGLDA is useful for inferring potential associations between lncRNAs and disease in practice.

## Discussions

Accumulating evidences have highlighted the important role of developing a powerful computational method to infer potential associations between lncRNAs and diseases, which can significantly help people to explore complex diseases at the molecular level and improve the quality of various disease diagnosis, therapy, prognosis and prevention. In this paper, we propose a novel computational method, TPGLDA, to identify the underlying lncRNA-disease associations by integrating experimentally verified gene-disease associations and lncRNA-disease associations. Compared with previous methods, we develop an lncRNA-disease-gene tripartite graph to better delineate the heterogeneity of coding-non-coding genes-disease associations. For the sake of better performance, we subsequently develop an effective resource allocation algorithm on the constructed lncRNA–disease-gene tripartite graph to rank candidates. In addition, TPGLDA can be applied to the isolated nodes by integrating lncRNA similarities and disease similarities. Our method firstly demonstrates its good performance by LOOCV experiment. Furthermore, the measures of AUC values and recall values within the top *k* ranking lists show that TPGLDA has powerful predictive ability to infer the largest number of positive samples. Finally, the analyses of case studies further demonstrate that TGPLDA is useful for identifying potential lncRNA-disease associations in practice.

The good results achieved by TPGLDA can be largely ascribed to following factors: firstly, motivated by cooperation between non-coding genes and coding genes in human diseases^14,18,22,55^, we effectively construct the associations among lncRNAs, diseases and genes, and develop an lncRNA-disease-gene tripartite graph to better delineate the heterogeneity of coding-non-coding genes-disease associations. The tripartite graph integrates a large number of disease-related genes as collaborative prediction of underlying association between lncRNAs and diseases, which enrich diseases information during the process of resource allocation^56^. Subsequently, the contributions of resource moved in both directions are taken into consideration by a consistence-based resource allocation algorithm^57^, which effectively reduces the unaware biases in resource allocation process^57,58^ and further improves TPGLDA’s predictive performance. Finally, by adopting different biological information including disease-related genes, lncRNA expression profile and disease semantic information in our method, potential candidates can acquire more information from other diseases and lncRNAs. In summary, TPGLDA shows a decent performance and complements the detection results of the existing approaches in inferring potential associations between lncRNAs and diseases. Nevertheless, the assessment measures are not sufficient to indicate a criticism of other computational approaches. Instead, different methods show the difference between whether considering the information of disease-related genes in the heterogeneous associations or not. As a novel computational method, it is anticipated that TPGLDA has potential value in biomedical research for comprehending the pathogenesis of diseases, which can further advance the quality of disease diagnostics, therapy, prognosis and prevention.

Despite the promising results achieved by TPGLDA, some limitations still should be acknowledged for further investigation. Firstly, TPGLDA depends on the tripartite graph topology and in consequence the incompleteness of the data may limit its performance. Therefore, it may be useful to further expand the method by integrating gene-lncRNA associations or additional biological information that have been successfully adopted in existing methods such as ncPred^26^, which can make our proposed method more accurate and reliable. Secondly, our method focuses on unweighted tripartite graph, it will be improved by a refined algorithm with accurately defined weights on lncRNA-disease as well as gene-disease^56^. Finally, the experimentally available lncRNA-disease associations are still comparatively small in number. With the continuous development of biotechnology, the performance of TPGLDA is expected to further increases when more experiment verified associations are available.

## Materials and Methods

### Human lncRNA–disease associations and gene-disease associations

The recent version of lncRNA-disease associations are downloaded from the LncRNADisease^35^ database which integrates 687 experimentally validated lncRNA–disease associations between 246 diseases and 369 lncRNAs. We further filter out diseases without Disease Ontology (http://disease-ontology.org/) information and lncRNAs without expression profiles in ArrayExpress^59^ (http://www.ebi.ac.uk/arrayexpress/), and eventually obtain 540 experimentally verified lncRNA-disease associations. This dataset is utilized as the gold standard dataset in the leave-one-out cross validation experiment and as the training dataset for inference of lncRNA–disease association^5^. Using the information of lncRNA-related diseases, we further collect 5212 gene–disease associations from DisGeNET database^24^ (http://www.disgenet.org/web/DisGeNET/) and construct a lncRNA-disease-gene tripartite graph, which includes 115 lncRNAs, 178 diseases and 1415 genes.

### LncRNA expression similarity

We obtain LncRNA expression profiles from ArrayExpress^59^ which contains more than 60000 expression profiles across 16 human tissues generated through RNA-Seq technology. Following previous approaches^5,60^, we calculate the lncRNA expression similarity (Supplementary Table S1) as the absolute Spearman correlation coefficient between the expression profiles of each lncRNA pair, and use matrix $\,SI{M}^{lnc}$ to denote the lncRNA expression similarity matrix between lncRNA ${l}_{i}$ and lncRNA $\,{l}_{j}$.

### Disease semantic similarity

Recently, disease semantic similarity has been used in predicting potential ncRNA-disease associations and its effectiveness has been demonstrated in previous studies^14,32,61^. In this paper, the disease semantic similarity is calculated in the same way as described in previous study^62^, in which a disease is represented as a directed acyclic graph (DAG) including all related annotation terms which can be obtained from the U.S. National Library of Medicine (MeSH, http://www.nlm.nih.gov/mesh). Based on their DAGs, the similarities between diseases are measured and the detailed calculations are illustrated in the DOSE package^63^. We eventually calculate the semantic similarities (Supplementary Table S2) among all diseases and the corresponding similarity matrix is denoted as $\,SI{M}^{dis}$.

### TPGLDA

Inspired by previous study using tripartite graph of users, items and tags for recommendation^56^, in this paper we first construct an lncRNA-disease-gene tripartite graph *T* (*L*, *D*, *G*, *E*), where *L* = {${l}_{1},{l}_{2},{l}_{3}\ldots {l}_{n}$}, *D* = {${d}_{1},{d}_{2},{d}_{3}\ldots {d}_{m}\}$ and *G* = {${g}_{1},{g}_{2},{g}_{3}\ldots {g}_{r}$} are the nodes set of *n* long non-coding RNAs, the nodes set of *m* diseases and the node set of *r* genes, respectively. *E* denotes the interactions (edges) set between nodes in *L* with *D* and *D* with *G*. The tripartite graph can also be represented by two adjacency matrices ${A}^{LD}={\{{a}_{ij}^{LD}\}}_{n\times m}\,$and ${A}^{GD}={\{{a}_{ij}^{GD}\}}_{m\times r}$, where ${a}_{ij}^{LD}=1$ if lncRNA ${l}_{i}$ is associated with disease ${d}_{j}\,$and otherwise ${a}_{ij}^{LD}$ = 0 indicating that the pair of lncRNA ${l}_{i}$ and disease ${d}_{j}$ is unknown association. Analogously, we set ${a}_{ij}^{GD}$ = 1 if disease ${d}_{j}$ is associated with gene $\,{g}_{i}$, otherwise 0.

**Figure 5:**
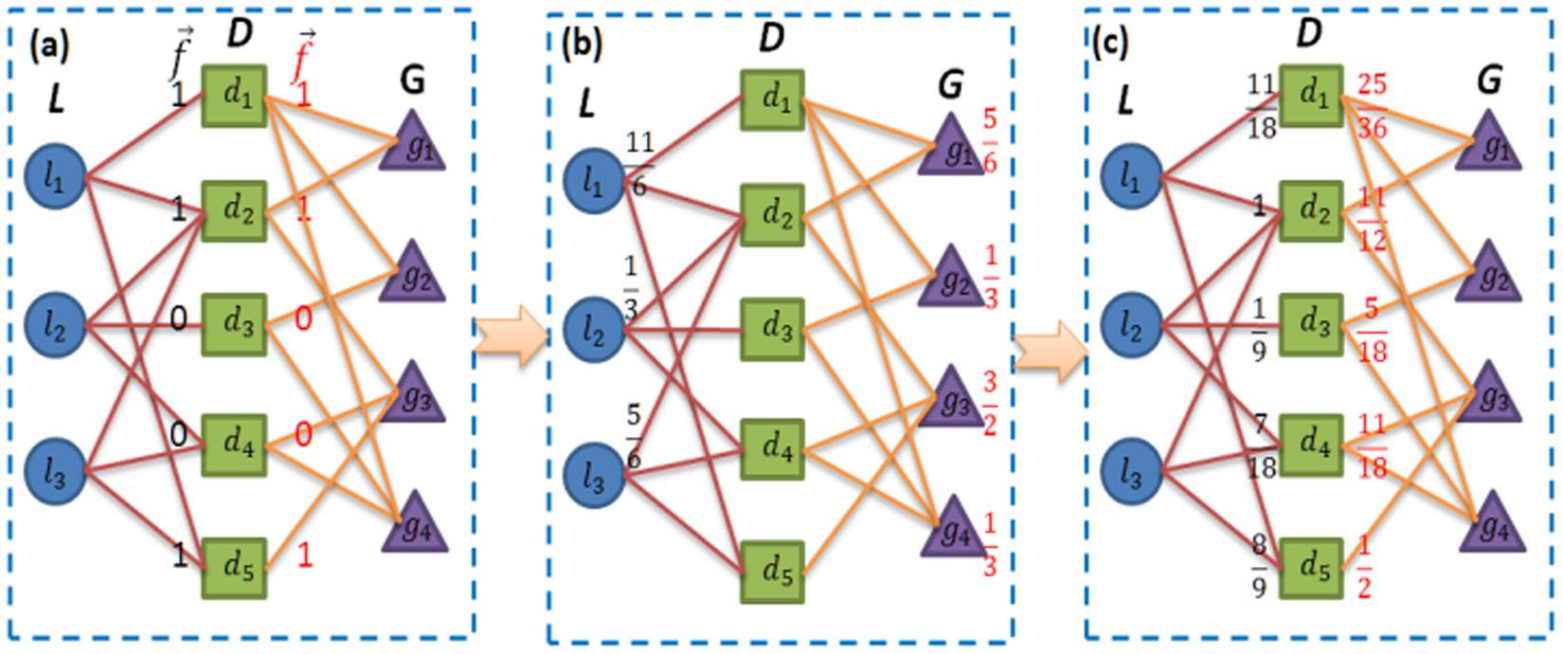
Operating principle of resource allocation in an lncRNA-disease-gene tripartite graph consisted of three lncRNAs, five diseases, and four genes. The blue circles, green squares and purple triangles represent lncRNAs in *L*, disease in *D* and genes in *G*, respectively. (**a**) For target lncRNA$\,{l}_{1}$, the initial resources $f({l}_{1})$(1, 1, 0, 0, 1) locate on$\,{d}_{i}$. (**b**) In the first step, each disease averagely distributes its resource to both sides of neighboring nodes based on the degree of each disease. (**c**) In the second step, the resources flow back to *D* from *L* and *G*, and final resource vector locate on *D* are $\mathop{\,{f}^{\text{'}}}\limits^{\rightharpoonup }(11/18,1,1/9,\,7/18,\,8/9)$ and$\mathop{\,{f}^{\text{'}\text{'}}}\limits^{\rightharpoonup }(25/36,11/12,\,5/18,11/18,1/2)$.

We model the prediction process of lncRNA–disease associations as resource allocation^56^ on the lncRNA-disease-gene tripartite graph. In order to help readers better understand the procedure of our model, a simple example of resource allocation in the tripartite graph is shown in Fig. 5. For a specific lncRNA $\,{l}_{i}$, the initial resources, located on disease ${d}_{j}$, is defined as:

6 $$f(\,{l}_{i})={a}_{ij}^{LD},\,j\,=\,1,\,2,\,\mathrm{..}.,\,m$$

The initial resource vector is then denoted as $f(\,{l}_{1})$ = (1, 1, 0, 0, 1) if we choose lncRNA ${l}_{1}$ as a target lncRNA (Fig. 5a). The resource allocation process in TGPLDA includes two steps. In the first step of the allocation, the initial resource is simultaneously allocated from nodes in *D* to those in *L* and *G*, respectively. In the second step, the resource is transferred back from nodes in *L* and *G* to the *D* nodes. We use the corresponding weight matrix $W$ = ${\{{w}_{ij}\}}_{n\times n}$ to represent the process of resource allocation between lncRNAs and diseases, as below:

7 $${w}_{ij}=\frac{1}{{k}_{l}({l}_{i})}\sum _{j=1}^{m}\frac{{a}_{ij}^{LD}{a}_{js}^{LD}}{{k}_{d}({d}_{j})}\,$$

where ${w}_{ij}$ is the contribution of resource moved from *j*-th node to *i*-th node in *L*, and can be described as the similarity^58^ between lncRNA ${l}_{i}$ and lncRNA ${l}_{j}$. ${k}_{l}({l}_{i})=\sum _{j=1}^{m}{a}_{ij}^{LD}$ is the number of related diseases for lncRNA $\,{l}_{i}$ which is called the degree of $\,{l}_{i}$. Similarly, ${k}_{d}({d}_{j})=\sum _{s=1}^{n}{a}_{js}^{LD}\,\,$represents the degree of $\,{d}_{j}\,\,$node in *D*. We further modify the resource allocation algorithm by considering the level of consistency between the contribution of resource moved in both directions^57^, which reflects the impact of co-selection (${l}_{i},{l}_{j}$) between the contribution of resource from ${l}_{i}$ to ${l}_{j}$ and the contribution of resource from ${l}_{j}\,\,$to $\,{l}_{i}$, as more consistency of two objects suggests higher similarity^58^. Accordingly, we define a consistence-based resource allocation to represent lncRNA-disease association as follows:

8 $$\,{w}_{ij}^{^{\prime} }={w}_{ij}+\frac{{w}_{ji}}{{\sum }_{{j}^{\text{'}}=1}^{m}{w}_{j\text{'}i}}\,$$

where $\,{w}_{ij}^{^{\prime} }$ denotes the sum of contribution from resource allocation between *i*-th node and *j*-th node in *L*. The corresponding weight matrix is then rewritten as $W^{\prime}$ = ${\{{w}_{ij}^{^{\prime} }\}}_{n\times n}$. Combining adjacent matrix $\,{A}^{LD}\,\,$and weight matrix $\,W^{\prime}$, the final resource $\mathop{\,\mathop{f\text{'}}\limits^{\rightharpoonup }}\limits^{\rightharpoonup }\,\,$located on *D* nodes is defined as:

9 $$\mathop{f^{\prime} }\limits^{\rightharpoonup }=W^{\prime} \times {A}^{LD}$$

With respect to resource allocation between genes and diseases, the same initial resource located on *D* nodes^56^ are allocated from nodes in *D* to nodes in *G* and then transferred back, and the final resource vector $\mathop{f\text{'}\text{'}}\limits^{\rightharpoonup }$ located on *D* nodes can be calculated as:

10 $$\mathop{f^{\prime\prime} }\limits^{\rightharpoonup }=\sum _{s=1}^{r}\frac{{a}_{js}^{GD}}{{k}_{g}({g}_{i})}\sum _{j=1}^{m}\frac{{a}_{ij}^{LD}}{k{^{\prime} }_{d}({d}_{j})}$$

where ${k}_{g}({g}_{i})\,$ = $\sum _{j=1}^{m}{a}_{ij}^{GD}$ represents the degree of gene ${g}_{i}$ in *G* and $k{^{\prime} }_{d}({d}_{j})$ = $\sum _{s=1}^{r}{a}_{js}^{GD}$ is the number of related genes for disease $\,{d}_{j}$. By weighting both $\mathop{f\text{'}}\limits^{\rightharpoonup }$ and $\,\mathop{f\text{'}\text{'}}\limits^{\rightharpoonup }$, the final resource score $\,Rscore$ used to measure potential lncRNA-related diseases are defined as follows:

11 $${R}_{score}=\gamma \,\mathop{f\text{'}}\limits^{\rightharpoonup }+(1-\gamma )\,\mathop{f\text{'}\text{'}}\limits^{\rightharpoonup }$$

where parameter $\gamma \in$[0, 1] is tunable and used to balance the contribution between lncRNAs and genes. In this paper, TPGLDA achieves the best prediction performance when $\,\gamma$ = 0.6. In fact, our method is robust and insensitive to the selection of $\gamma$(see Supplementary Fig. S1).

The inference of the isolated node is implemented by following procedure proposed in previous study^27^, which can be summarized as follows. First, we calculate the similarity $SIM({s}_{new},{s}_{i})$ between an isolated node (e.g. a new lncRNA) and its neighbors, which is calculated by lncRNA expression similarity for an isolated lncRNA or disease semantic similarity for an isolated disease. Second, we calculate the interaction profile $\,{S}_{new}$ by the following form:

12 $${S}_{new}=\sum _{i=1}^{{n}_{new}}(SIM({S}_{new},{S}_{i}))\cdot {a}_{i}$$

where $\,{a}_{i}$ is an interaction profile vector and $\,{S}_{new}$ is used to reflect potential relationships between the isolated node and diseases by considering its neighbors interactions with diseases^27^, which is then integrated into tripartite graph for further resource allocation.

## Electronic supplementary material

Supplementary Information

Supplementary Table S1

Supplementary Table S2

Supplementary Table S3
