# Supplementary material for: TPGLDA: Novel prediction of associations between lncRNAs and diseases via lncRNA-disease-gene tripartite graph
Source: Sci Rep. 2018 Jan 18;8:1065. doi: 10.1038/s41598-018-19357-3 (PMC5773503; doi:10.1038/s41598-018-19357-3)
Supplement: Supplementary file 1 — Supplementary Information [file 41598_2018_19357_MOESM1_ESM.pdf]

TPGLDA: Novel prediction of associations between lncRNAs and diseases via lncRNA-disease-gene tripartite graph

Liang Ding[1,+], Minghui Wang [1,2,+,*], Dongdong Sun[1] and Ao Li [1,2]

[1]School of Information Science and Technology, University of Science and Technology of China, Hefei AH230027, China

[2]Centers for Biomedical Engineering, University of Science and Technology of China, Hefei AH230027, China

*Correspondence and requests for materials should be addressed to M.W.(email: mhwang@ustc.edu.cn)

[+] These authors contributed equally to this work

Supplementary Information

Supplementary Figure 1. Prediction performance affected by γ

Supplementary Table 1. The results of the lncRNA expression similarity

Supplementary Table 2. The calculated results of the semantic similarity among all diseases.

Supplementary Table 3. The predictions of potential lncRNA-disease associations by TPGLDA.

Supplementary Table 4. The top 10 prediction results for colorectal cancer, bladder cancer, breast cancer and gastric cancer.

Supplementary Table 5. The top 20 potential lncRNA-disease associations ranked by TPGLDA and confirmation for their associations by related databases or literatures. 12 of the top 20 disease–lncRNA associations have been confirmed.

Supplementary Table 6. Friedman rank sum test used to determine the statistical significance in the performance improvement of TPGLDA compared to KRWRH and LRLSLDA on our gold dataset.

Supplementary Table 7. Comparison between TPGLDA and ncPred using one of the dataset in ncPred

Supplementary Table 8. Friedman rank sum test used to determine the statistical significance in the performance improvement of TPGLDA compared to ncPred on Chen's dataset.

Supplementary Table 9. Running time comparison of TPGLDA, KRWRH and LRLSLDA.

Figure

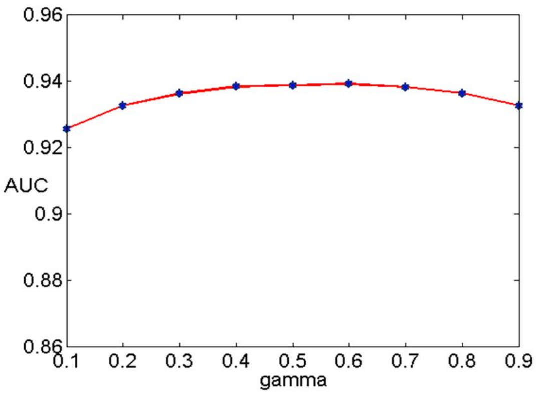

Figure 1. Prediction performance affected by γ.

Table

LncRNA	TPGLDA's rank	LncRNA	TPGLDA's rank
Colorectal Cancer			
TUG1	1	BC040587	6
GHET1	2	CDKN2B-AS1	7
SPRY4-IT1	3	LINC00261	8
DNM3OS	4	SOX2-OT	9
MINA	5	HIF1A-AS2	10
Bladder Cancer			
PVT1	1	BANCR	6
CCAT2	2	CDKN2B-AS1	7
CCAT1	3	LSINCT5	8
HULC	4	RPL34-AS1	9
TUSC7	5	MINA	10
Breast Cancer			
CHL1-AS2	1	MINA	6
BANCR	2	TUSC7	7
HULC	3	DNM3OS	8
CCAT1	4	MNX1-AS1	9
TUG1	5	GHET1	10
Gastric Cancer			
TUG1	1	HOTTIP	6
XIST	2	MNX1-AS1	7
SNHG16	3	NPTN-IT1	8
KCNQ1OT1	4	HIF1A-AS1	9
NEAT1	5	PANDAR	10

Table S4. The top 10 prediction results for colorectal cancer, bladder cancer, breast cancer and gastric cancer.

**Rank**	**Diseases**	**lncRNAs**	**Evidences**(PMID)	**Description**
1	epithelial ovarian cancer	H19	Unconfirmed	Unconfirmed
2	epithelial ovarian cancer	MALAT1	Unconfirmed	Unconfirmed
3	epithelial ovarian cancer	CDKN2B-AS1	Unconfirmed	Unconfirmed
4	osteosarcoma	H19	PMID:27008415	literature
5	osteosarcoma	HOTAIR	PMID:25728753	Lnc2Cancer
6	epithelial ovarian cancer	MEG3	PMID:24859196	Lnc2Cancer
7	osteosarcoma	CDKN2B-AS1	PMID:26408699	Lnc2Cancer
8	osteosarcoma	MEG3	PMID:26823857	Lnc2Cancer
9	small cell lung cancer	H19	Unconfirmed	Unconfirmed
10	cervical cancer	CDKN2B-AS1	PMID:22487937, PMID:27008415	Lnc2Cancer, literature
11	epithelial ovarian cancer	GAS5	Unconfirmed	Unconfirmed
12	non-small cell lung cancer	H19	PMID:26482621	Lnc2Cancer
13	renal cancer	MALAT1	PMID:26461224	Lnc2Cancer
14	cervical cancer	PVT1	PMID:27272214, PMID:27232880	Lnc2Cancer, literature
15	epithelial ovarian cancer	PVT1	Unconfirmed	Unconfirmed
16	osteosarcoma	PVT1	PMID:27813492	literature
17	psoriasis	CDKN2B-AS1	Unconfirmed	Unconfirmed
18	renal cancer	H19	PMID:25866221	literature
19	osteosarcoma	GAS5	Unconfirmed	Unconfirmed
20	renal cancer	HOTAIR	PMID:24616104	Lnc2Cancer

Table S5. The top 20 potential lncRNA-disease associations ranked by TPGLDA and confirmation for their associations by

Dataset	Friedman χ 2	p-Value
Our golden dataset	133.8	$< 2.2\times10^{-16}$

Table S6. Friedman rank sum test used to determine the statistical significance in the performance improvement of TPGLDA compared to KRWRH and LRLSLDA on our golden dataset.

Dataset	AUC	
Chen et al. (2013)	TPGLDA	ncPred
	0.7586±0.0306	0.7566±0.0218

Table S7 Comparison between TPGLDA and ncPred using one of the dataset in ncPred.

Dataset	Friedman χ 2	p-Value
Chen et al. (2013)	1.2	0.2733

Table S8. Friedman rank sum test used to determine the statistical significance in the performance improvement of TPGLDA compared to ncPred on Chen et al dataset.

Methods	TPGLDA	KRWRH	LRLSLDA
Running time/seconds	0.61	28	0.22

Table S9. Running time comparison of TPGLDA, KRWRH and LRLSLDA.
